# Personality disorders: the impact of severity on societal costs

**DOI:** 10.1007/s00406-023-01715-6

**Published:** 2023-11-22

**Authors:** Carl-Aksel Sveen, Geir Pedersen, Benjamin Hummelen, Elfrida Hartveit Kvarstein

**Affiliations:** 1 Vestre Viken Hospital Trust, Drammen, Viken Norway; 2 Institute of Clinical Medicine, University of Oslo, Oslo, Norway; 3 Network for Personality Disorder, Section for Personality Psychiatry and Specialized Treatments, Department for National and Regional Functions, Division of Mental Health and Addiction, Oslo University Hospital, Oslo, Norway; 4 Department of Research and Innovation, Division of Mental Health and Addiction, Oslo University Hospital, Oslo, Norway; 5 Section for Personality Psychiatry and Specialized Treatments, Department for National and Regional Functions, Division of Mental Health and Addiction, Oslo University Hospital, Oslo, Norway

**Keywords:** Personality disorders, Societal costs, Severity, Cost-of-illness

## Abstract

Personality disorders (PDs) are associated with high levels of societal costs. However, previous research has found limited or no evidence of unique contributions of individual PD categories on the overall level of societal costs. Recent research supports the validity of PD as a dimensional construct, and PD severity may be a better predictor of societal costs than specific PD categories. The aim of this study was to explore if PD severity could predict the level of societal costs among treatment-seeking patients with PDs, while controlling for the impact of comorbid mental health and substance use disorders. Four different severity indicators were explored: the number of PDs, the total number of PD criteria, the number of BPD criteria, and the Level of Personality Functioning Scale (LPFS) from the alternative model in DSM-5. Participants (*n* = 798/794) were retrieved from the quality register of the Norwegian Network for Personality Disorders for the period 2017–2020. Societal costs were assessed using a structured interview covering the six-month period prior to assessment. Diagnoses and diagnostic criteria were determined using a semi-structured diagnostic interview (SCID-5-PD and M.I.N.I), and the LPFS was assessed by the LPFS-Brief Form 2.0 (LPFS-BF 2.0) questionnaire. Statistics included multiple regression analyses. None of the severity indicators were significant predictors of overall societal costs among treatment-seeking patients, and effect sizes were small.

## Introduction

High levels of societal costs incurred by patients with personality disorders (PDs) have been demonstrated in several studies [15, 18, 19, 49, 54, 61, 63, 67, 68]. A recent Norwegian study, with a large sample of treatment-seeking patients, investigated the individual contribution of specific PD categories on societal costs, as well as its two main components: health service costs and productivity loss [60]. The study demonstrated that specific type of PD did not discern differences in the overall level of societal costs. However, for the underlying cost components there were some notable findings: Only borderline PD (BPD) had a unique contribution to health service costs, while BPD, avoidant PD, and unspecified PD were all associated with enhanced productivity loss. In spite of these significant results, the effect sizes and levels of explained variance were small. A possible deficiency in the clinical utility of the current categorical diagnostic system could be a possible explanation of the weak association between specific PD categories and societal costs [7, 36], and a dimensional construct of PD severity might be a better predictor of societal costs than PD categories. To our knowledge, no studies have as of yet explored the impact of dimensional models of PD severity on societal costs.

The determination of illness severity has important clinical implications, and severity affects decisions to seek treatment, the type and intensity of treatment, and whether to continue or stop treatment [77]. In recent years, research on PD severity has been increasing, in line with a more dimensional approach to PDs in the research literature [31]. However, the research literature reveals a multitude of conceptualizations and possible measures of severity [2], 3, 6, 9, 11, 14, 77. According to Zimmerman et al. [77], these include the number of criteria met for specific PDs, among which BPD is most frequently used, or total criteria across PDs [3, 6, 9, 14, 33, 75], the number of PD diagnoses overall [75, 76], the number of PD clusters involved [62, 75], the type of PD (a severity hierarchy among PD types) [30], level of maladaptive functioning [11, 14, 65, 71], and the extent of comorbidity with other mental health and substance use disorders (previously called Axis I disorders in DSM-IV) [9, 33].

These developments are reflected in new revisions of both classification systems for mental health disorders. The current *Diagnostic and Statistical Manual of Mental Disorders* (5th edition, DSM-5) classification system still uses the same categorical classification of PDs as DSM-IV (Section II), but an Alternative Model of PDs (AMPD) was included in Section III (Emerging Measures and Models) among concepts requiring additional study [1]. The AMPD is a hybrid dimensional/categorical model representing PDs as core impairments in personality functioning in form of a 5-point rating scale, the Level of Personality Functioning Scale (LPFS), and specific configurations of problematic personality traits [37, 38]. Correspondingly, the importance of severity in classifying PDs is reflected in the implementation of a five-level severity scale of PD in the *International Classification of Diseases* (11th edition, ICD-11), in combination with an optional combination of five possible trait domain specifiers, and an additional borderline pattern specifier [4, 70].

Based on the general change in the conceptualization of PDs toward a more dimensional model, both in the research literature and in the classification systems, the aim of the present study was to investigate how well four of the different severity indicators could predict societal costs in separate regression analyses. Three of these severity indicators were based on the Section II model of DSM-V, i.e., the number of PDs, the total number of PD criteria, and the number of BPD criteria, whereas the last severity indicator was based on the AMPD, i.e., the A criterion, or LPFS. Other mental health and substance use disorders were included in all regression analyses as covariates to control for possible confounding effects. Supplementary analyses included the severity indicators’ contributions to the components of societal costs, i.e., health service costs and productivity loss. Furthermore, as the present study used the same sample of treatment-seeking patients as the abovementioned Norwegian study of the relative contribution of individual PD categories on societal costs [60], the contrasting conceptualizations of distinct PD categories and dimensional measures of PD severity could be compared.

## Methods

### Setting and recruitment

The present study was based on data for the period 2017–2020 retrieved from the quality register of the Norwegian Network for Personality Disorders (Network), a nationwide clinical research collaboration [29, 43]. It included 15 different outpatient treatment units within the Network, which offer specialized treatment for adult patients with a variety of PDs or clinically relevant, subthreshold personality difficulties. Patients were referred to specialized PD treatment from regular mental health outpatient clinics, where an initial assessment of patients referred from general practitioners was performed. Patients with comorbid psychosis, bipolar I disorder, autism, mental disability, and severe substance use disorders are not considered eligible for the PD-treatment programs, but in practice, a minor proportion was nonetheless referred. The treatment units comprise multidisciplinary teams with different healthcare professionals including psychiatrists, psychologists, psychiatric nurses, social therapists, and occupational therapists. All units within the Network follow the same assessment procedures, using standard evaluation instruments and diagnostic interviews. Treatment approaches include specialized BPD programs (Mentalization-based treatment, Dialectical Behavioral Therapy, Schema-focused therapy) as well as psychodynamic group therapy, metacognitive interpersonal therapy, art therapy, body awareness therapy and groups focusing on psychoeducation [61].

### Participants

Two different samples were used for the four separate regression analyses: One common sample for the number of PDs, the total number of PD criteria, and the number of BPD criteria, and a separate sample for the LPFS model from the AMPD.

#### Sample I

The sample which was used to study the number of PDs and the total number of PD criteria included 798 patients who had completed the cost interview (a specific interview of health and welfare service use and occupational activity), and were assessed for both PDs and comorbid other mental health and substance use disorders. In the sample, 24.6% were male and 75.4% were female, and the mean age was 30.0 (SD = 8.9, range 18–63 years). Table 1 presents the distribution of PD diagnoses in the sample.

**Table 1:** The distribution of personality disorders

	Frequency	Percent
Unspecified	102	12.8
Schizoid	9	1.1
Schizotypal	3	0.4
Paranoid	68	8.5
Antisocial	19	2.4
Narcissistic	3	0.4
Borderline	267	33.5
Histrionic	5	0.6
Avoidant	291	36.5
Dependent	41	5.1
Obsessive–compulsive	58	7.3

*N* = 798. As patients can be diagnosed with more than one diagnosis of PD, the percentages will add up to more than 100%

#### Sample II

The sample used to study the LPFS model included 794 patients who had completed the cost interview, were assessed for comorbid other mental health and substance use disorders, and had completed a questionnaire for assessing LPFS. Sample I and II have 93% overlap, as they share 745 participants. The 7% discrepancy is due to the fact that some patients in the Network’s quality register who were assessed for PDs had not completed the questionnaire for assessing LPFS, and vice versa. In this sample, 23.9% were male and 76.1% were female, and the mean age was 30.04 (SD = 8.8, range 18–63 years).

#### Other mental health and substance use disorders (covariates) in the samples

Nearly, all (Sample I: 94.7%, Sample II: 94.2%) of the assessed patients were given at least one other mental health or substance use diagnosis (Sample I: mean = 2.02, SD = 1.30, Sample II: mean = 1.98, SD = 1.28). Most individual diagnoses were aggregated into categories, and the five most frequent categories were used as covariates in the regression analyses; Mood disorders, anxiety disorders, substance use disorders, eating disorders, and PTSD. The omitted diagnoses (somatoform disorder, dissociative disorder, ADHD, psychosis disorders, and autism spectrum disorder) had too few incidents (< 8%) to warrant inclusion as covariates in the analyses. Table 2 describes the number of patients in the different other mental health and substance use categories in both samples.

**Table 2:** Categories of other mental health and substance use disorders

	Sample I		Sample II	
	Frequency	Percent	Frequency	Percent
Mood disorders	560	70.2	550	69.3
Anxiety disorders	415	52.0	405	51.0
PTSD	107	13.4	107	13.4
Substance use disorders	83	10.4	71	8.9
Eating disorders	73	9.1	69	8.7

Sample I: *N* = 798 and Sample II = 794. As patients can be diagnosed with more than one diagnosis of other mental health and substance use disorders, and thereby could be included in more than one category, the percentages will add up to more than 100%

### Diagnostic assessment: the number of PDs, the total number of PD criteria, and the number of BPD criteria

The measures of three of the severity indicators, the number of PDs, the total number of PD criteria, and the number of BPD criteria, as well as the covariates of other mental health and substance use disorders, were based on standardized, semi-structured diagnostic interviews: The Structured Clinical Interview for DSM-5 Personality Disorders for PD (SCID-5-PD) [16], and the Mini International Neuropsychiatric Interview (M.I.N.I.) [52, 53] for other mental health and substance use disorders. Table 3 presents the number of PDs per patient in the sample, whereas Table 4 presents the distribution of SCID-5-PD criteria in the sample. Table 5 presents the number of BPD criteria per patient in the sample.

**Table 3:** The number of personality disorders for each patient

Number of PDs	Frequency	Percent
0	159	19.9
1	474	59.9
2	116	14.5
3	38	4.8
4	9	1.1
5	2	0.3
Total	798	100

**Table 4:** The distribution of SCID-5-PD criteria

*N*	Minimum	Maximum	Mean	Median	Mode	Std.dev
798	0	50	12	11	8	7.20

**Table 5:** The number of BPD criteria per patient

Number of criteria	Frequency	Percent
0	170	21.3
1	124	15.5
2	93	11.7
3	78	9.8
4	66	8.3
5	80	10.0
6	71	8.9
7	62	7.8
8	42	5.3
9	12	1.5
Total	798	100

267 patients were diagnosed with BPD, as they fulfilled five or more criteria

Diagnostic inter-rater reliability, using the SCID-5-PD and M.I.N.I., was not directly investigated in this study. However, several measures were undertaken to address possible reliability issues. Within the Network, diagnostic assessments were performed in each unit by clinical staff who had received systematic training in diagnostic interviews and principles of the LEAD-procedure (Longitudinal, Expert, All-Data), [42, 56]. This means that diagnoses were based on all available information including referral letters, self-reported history and complaints, and overall clinical impression, in addition to the diagnostic interviews. All diagnoses were set or evaluated by a specialist in psychiatry or clinical psychology. In the study period, local training courses/workshops focusing on understanding and assessment of PDs, associated comorbidity, and use of structured interviews were conducted by an experienced psychiatrist (last author) at all units to ensure clinical competence and calibrate diagnostic evaluation. A total of 29 local workshops were held within the study period in addition to shorter clinical discussions on request [60]. Furthermore, in a former study within the Network, using the Structured Clinical Interview for DSM-IV (SCID-II – the previous version of SCID-5-PD), reliability was investigated and acceptable diagnostic reliability was indicated [17].

As first defined in the DSM-III (APA, 1980), PD-not otherwise specified (PD-NOS) is indicated when the general criteria for PD are fulfilled, but criteria are below the threshold of any specific PD. The diagnosis is either set directly by the clinicians or set by the researchers according to a given set of criteria. The operationalization of PD-NOS lacks precision, and former studies have suggested cut-offs ranging from 5 to 11 fulfilled PD criteria across categories [13, 41, 42, 66, 72]. In the current study, based on SCID-5-PD and DSM-5 terminology, we chose to categorize patients with eight or more fulfilled PD criteria and no specific PDs as unspecified PD (if not already given the diagnosis by the clinicians) [61].

### Assessment of the LPFS model

LPFS reflects core dimensions of personality pathology, involving impairments in self and interpersonal functioning, with self-functioning represented by the domains of identity and self-direction, and interpersonal functioning represented by empathy and intimacy [79]. All four domains are described along a continuum that ranges from healthy functioning (level = 0), to extreme impairment (level = 4) [37, 38]. Each of these components are further subdivided into three indicators (subdomains), i.e., 12 indicators altogether, and the LPFS thus identifies five levels of functioning for each of these 12 indicators, offering a severity index for personality pathology [24, 69].

Since its publication, several instruments for assessing the LPFS have been developed, and in the present study we used the second version of the LPFS-Brief Form (LPFS-BF 2.0) questionnaire [69]. The LPFS-BF is a 12-item self-report, each item representing one of the 12 indicators of the LPFS, yielding a global estimate of impairment related to personality functioning [25, 69]. Initially developed as a quick screening tool related to the LPFS, this instrument is now included in the standard set of patient-reported outcomes for PD [47]. The first version was subsequently revised, by rewording some items and introducing a 4-point Likert scale from 0 (completely untrue) to 3 (completely true). The severity index will thus have a possible range of 0–36 points [69]. The LPFS-BF 2.0 showed acceptable construct validity and psychometric properties [5, 48, 69]. Initially, there were some concerns that the LPFS-BF yielded a two-factor solution [69, 78]. However, results of recent bifactor studies have indicated that the LPFS, as assessed by the LPFS-BF, can be considered as essentially unidimensional [48, 79]. Table 6 presents the distribution of LPFS-BF 2.0 scores in the sample.

**Table 6:** The distribution of LPFS-BF 2.0 total scores

*N*	Minimum	Maximum	Mean	Median	Mode	Std.dev
794	1	36	18.46	19	20	6.67

### Cost measures

Societal costs are the sum of direct and indirect costs. Direct costs cover all actual costs of healthcare utilization, e.g., general practitioner visits, psychotherapeutic treatment, medication, inpatient treatment (both somatic and mental health). Indirect costs cover the lost productivity due to suffering from PD. Intangible costs, i.e., the psychological pain experienced by people with PDs, are not included in societal costs in this study, as such costs are very difficult to measure [28]. Hence, the societal costs in this study are the sum of direct healthcare costs and productivity loss. Calculations of healthcare costs and productivity loss for the total period of 6 months prior to evaluation were estimated using a bottom-up approach [28], i.e., taking the individual patients’ reported health service use and degree of absenteeism from the labor market, and multiplying it with the estimated unit cost of each specific cost element [60].

Clinicians performed the cost interview as a part of the pretreatment assessment, collecting data for the six-month period prior to assessment. Questions on health service use included: (1) general practitioner (GP) visits, (2) emergency health services (psychiatric emergency helpline, emergency room, psychiatric outpatient emergency service, and ambulant emergency service), (3) hospitalization (admission to medical hospital, admission to psychiatric hospital, admission to addiction clinics, and day-patient care), (4) outpatient treatment at mental health centers (individual- or group therapy), and (5) pharmacological treatment. The participants were also asked to which degree they were employed the last six months (range 0–6) [60].

All unit costs were measured in €, yearend 2018. Unit cost for GP was estimated based on a public report that estimated the total cost of all GPs [27] in 2017, adjusted by the official consumer price index (CPI) [57], divided by the total number of consultations by GPs during 2018 [58]. Unit cost for psychiatric emergency helpline was calculated based on the annual report 2018 from “Mental Helse”, a typical helpline provider in Norway [35]. The total cost of the service was divided by the total number of telephone calls (answered), chat-service and mail service. Emergency room unit cost was set to the price for same day consultation with specialist medical doctor at a private healthcare center in Oslo [40]. Psychiatric emergency outpatient service was set at the same unit cost as standard outpatient consultation, while emergency consultation at the patients home out of an outpatient clinic were given an ambulatory fee add-on [20]. Unit costs related to treatment at outpatient mental health centers, medical and psychiatric hospitals, addiction clinics, and day-patient care were obtained from reports published by the Norwegian government [21, 22]. Calculations of medication unit costs were based on information from the Norwegian Medicines Agency, and cost per daily dose of typical drugs per medication class were used to calculate cost per month [34].

The human capital approach was used to calculate the productivity loss, as most cost-of-illness studies have used the human capital approach to estimate productivity loss [46, 64]. This method measures lost productivity as the patients’ absence from work due to illness, valued at the market wage. As the patients did not report their individual gross income, and the marked wage of patients with PDs is not available in public registers, the patients’ unit cost had to be estimated. As many patients with PDs struggle to stay in the workforce and achieve higher levels of education, the average monthly wage for the total population probably is an overestimation of the wage-level of patients with PDs (only 12% of both samples report they have been in ordinary employment during the whole 6 months, while 73% of both samples report no connection to the labor marked during the same period). The unit cost of lost productivity was, thus, set to be equal to the average monthly sickness benefit [39], which is 58% of the average monthly wage in Norway [59].

All unit costs, mean health service costs, mean productivity loss, and mean societal costs in the period six months prior to assessment are reported in detail by Sveen and colleagues in their cost-of-illness study of treatment-seeking patients with PDs, using data from the quality register for the same period as the present study [61].

### Ethics

All participating patients from each treatment unit gave their written consent to use anonymous clinical data for research purposes. Anonymized data were collected and transferred to the quality register. The collection procedures were approved by a local data protection officer at each contributing unit. Data security procedures for the quality register were approved by the data protection officer at the research center of the Network at Oslo University Hospital. Because the data were anonymous, formal approval from the Norwegian State Data Inspectorate and Regional Committee for Medical Research and Ethics was not required [61].

### Statistical analysis

Four separate multiple regression main effect analyses, one for each severity indicator, were performed in order to investigate how well each of them could predict societal costs, while controlling for the effects of the five categories of comorbid other mental health and substance use disorders. All four regression models thus included six independent variables. Due to the exploratory nature of the current investigation, no general adjustments for multiple comparisons was strictly required, and an alpha level of 0.05 was used to determine statistical significance for all analyses [10]. Tables 7, 8, 9 and 10 present exact *p* values, and power analyses were conducted post hoc. The correlation matrix between all the independent variables as well as the Tolerance and Variance Inflation Factor (VIF) coefficients gave no indication of a multicollinearity problem in any of the models.

**Table 7:** Regression model 1: Number of PDs as severity indicator

Independent variable	*p*	*β*
Number of PDs	0.672	0.015
Mood disorders	0.742	− 0.012
Anxiety disorders	0.828	0.008
Substance use disorders	0.416	0.029
Eating disorders	< 0.001	0.130
PTSD	0.136	0.053
		*R*^2^
Model 1	0.010	0.021

N = 798. *p* values (independent variables and *F* test of overall model), beta weights, and *R*^2^ are presented

**Table 8:** Regression model 2: Total number of PD criteria as severity indicator

Independent variable	*p*	*β*
Total number of PD criteria	0.765	0.011
Mood disorders	0.733	− 0.012
Anxiety disorders	0.812	0.009
Substance use disorders	0.419	0.029
Eating disorders	< 0.001	0.130
PTSD	0.138	0.053
		*R*^2^
Model 2	0.010	0.021

*N* = 798. *p* values (independent variables and *F*-test of overall model), beta weights, and *R*^2^ are presented

**Table 9:** Regression model 3: Number of BPD criteria as severity indicator

Independent variable	*p*	*β*
Number of BPD criteria	0.948	− 0.002
Mood disorders	0.724	− 0.013
Anxiety disorders	0.764	0.011
Substance use disorders	0.389	0.031
Eating disorders	< 0.001	0.130
PTSD	0.123	0.055
		*R*^2^
Model 3	0.010	0.021

*N* = 798. *p* values (independent variables and *F* test of overall model), beta weights, and *R*^2^ are presented

**Table 10:** Regression model 4: LPFS-BF 2.0 total scores as severity indicator

Independent variable	*p*	*β*
LPFS-BF	0.900	0.004
Mood disorders	0.625	− 0.017
Anxiety disorders	0.984	0.001
Substance use disorders	0.768	− 0.010
Eating disorders	< 0.001	0.138
PTSD	0.104	0.057
		R^2^
Model 4	0.008	0.022

*N* = 794. *p* values (independent variables and *F*-test of overall model), beta weights, and *R*^2^ are presented

Societal costs data in the present study were non-normally distributed. Most patients had similar health service costs, but a small proportion of patients had very high costs due to inpatient admissions. As many as 73% of the patients had been out of the workforce during all 6 months, incurring a large productivity loss, while only 12% had no productivity loss. The residuals were non-normally distributed as well. They were not improved by preliminary trials of log transformations. However, when the sample size is sufficiently large, as in the present sample, the Central Limit Theorem ensures that the distributions of parameter estimates will approximate normality when the errors are independent and identically distributed with finite variance, regardless of the shape of the population distribution [44, 45, 50]. Supplementary subgroup analyses of health service costs and productivity loss were performed to further explore the impact of the severity measures on costs. As the residuals of the health service costs model approximated the normal distribution when it was log transformed, but no improvement of the residuals of the productivity loss model was made by such a transformation, a log transformed model was used in the analysis of health service costs, because a transformation which could improve the normality of the residuals significantly is recommended, also when the sample size is large [44]. Statistical analyses were performed using SPSS version 29, except for the power analyses, which were performed using the R package «pwr» (version 1.3-0).

## Results

In Tables 7, 8, 9 and 10, the main effects of the respective, multiple regression analyses regarding number of PDs, total number of PD criteria, number of BPD criteria, and LPFS-BF 2.0 total score, with the other mental health and substance use disorder covariates on societal costs are displayed.

### Model 1: Number of PDs

The number of PDs was not a significant predictor of societal costs, and the effect size (beta weight) was small (0.015). Eating disorders was the only significant covariate in this regression model. The proportion of explained variance was 2.1% (*R*^2^ = 0.021), and the overall model was significant (*p* = 0.010). Given the sample size, a significance level of 0.05, six independent variables, and the level of *R*^2^, the statistical power of this regression model was high (0.89). In supplementary regression analyses of the components of societal costs, including the covariates, the number of PDs was neither a significant predictor of health service costs nor productivity loss.

### Model 2: Total number of PD criteria

The total number of PD criteria was not a significant predictor of societal costs, and the effect size was small (0.011). Eating disorders was the only significant covariate. The proportion of explained variance by this regression model was 2.1% (*R*^2^ = 0.021), and the overall model was significant (*p* = 0.010). Given the sample size, a significance level of 0.05, six independent variables, and the level of *R*^2^, the statistical power of this regression model was high (0.89). Supplementary regression analyses of the components of societal costs, including the covariates, showed that the total number of PD criteria was neither a significant predictor of health service costs nor productivity loss.

### Model 3: Number of BPD criteria

The number of BPD criteria was not a significant predictor of societal costs, and the effect size was close to zero (0.002). Having an eating disorder diagnosis was the only significant covariate. The proportion of explained variance by this regression model was 2.1% (*R*^2^ = 0.021), and the overall model was significant (*p* = 0.010). Given the sample size, a significance level of 0.05, six independent variables, and the level of *R*^2^, the statistical power of this regression model was high as well (0.89). Supplementary regression analyses of the components of societal costs, including the covariates, showed that the number of BPD criteria was a significant predictor of health service costs (*p* = 0.008), with a beta weight of 0.096, but not a significant predictor of productivity loss.

### Model 4: Total score of LPFS-BF 2.0

The level of LPFS-BF 2.0 total score was not a significant predictor of societal costs, and the effect size was very small (0.004). Having an eating disorder diagnosis was the only significant covariate. The proportion of explained variance by this regression model was 2.2% (*R*^2^ = 0.022), and the overall model was significant (*p* = 0.008). Given the sample size, a significance level of 0.05, six independent variables, and the level of *R*^2^, the statistical power of this regression model was high (0.91). In supplementary regression analyses of the components of societal costs, including the covariates, the level of LPFS-BF 2.0 total score was a significant but weak predictor of health service costs (*p* = 0.035), with a beta weight of 0.075. The total score of LPFS-BF 2.0 was not a significant predictor of productivity loss.

## Discussion

Our main finding was that none of the indicators of PD severity were significant predictors of societal costs in this sample of treatment-seeking patients. The effect sizes were small, and the high level of statistical power in all four models underscores the low probability of false-negative findings. Furthermore, the fact that all four of the severity indicators rendered similar results, in spite of their different operationalizations (three were based on diagnostic interviews, while one was based on a self-report questionnaire), further substantiates that differences in severity levels measured by these indicators explain little variation in societal costs within this patient group.

These findings are somewhat surprising, as one would generally expect that more severe personality dysfunction would lead to a more extensive use of healthcare resources and a lower level of workforce participation, i.e., higher levels of societal costs. However, possible selection bias may have had an influence on the results. The study investigates patients who are referred to treatment, and indications for PD treatment may typically be lack of work functioning and/or a high symptom burden leading to use of a range of health services [8, 12, 32]. As many as 75% of the patients were outside the labor market during the whole 6-month period before diagnostic assessment, incurring a large productivity loss, while only 10.3% had no productivity loss. Furthermore, health service costs (the other component of societal costs), were moderate for most patients, while a small proportion of patients incurred very high health service costs due to extensive use of inpatient treatment [61]. The bulk of treatment-seeking patients may, thus, have been quite homogeneous with respect to features captured in the cost parameters, as the level of societal costs were generally high, with a limited degree of variability, for most patients in this group, possibly resulting in a relatively low level of explained variance in the regression models.

As the aggregation of cost components to overall societal costs may have disguised possible nuances in the impact of PD severity, we also performed supplementary subgroup analyses with health service costs and productivity loss, respectively, as dependent variables. These analyses indicated that the four measures of PD severity generally had limited impact on the component level as well. Nonetheless, although all four severity indicators were non-significant for the productivity loss subcomponent, the LPFS-BF 2.0 total score and number of BPD criteria were significant predictors of health service costs. This finding could be explained by the strong association between the LPFS-BF 2.0 and BPD. A recent study from the Network demonstrated that among all specific PD criteria, the number of BPD criteria had the highest correlation with LPFS-BF 2.0 total score [48], and previous research suggests that BPD could be considered more like a global index of personality pathology than a distinct PD type [51, 73, 74]. Furthermore, in a study from the Network based on the same sample as the current study, BPD had significantly higher health service costs than all other PDs [60]. Thus, it is possible that LPFS-BF 2.0 associations with health service costs found in the present study reflects its strong association with the BPD construct.

It may be somewhat surprising that the two other severity measures, i.e., the total number of PD criteria and the number of PDs, did not demonstrate a significant relationship between their increasing levels and higher health service costs, as both are considered as possible expressions of PD severity in the literature [62, 75, 77]. However, a possible explanation could be that both the total criterion-count and total diagnoses-count include a heterogeneity of PD features. For instance, it could be that patients with either many fulfilled PD criteria or a high number of comorbid PDs have traits that both tend to increase costs (self-harm that leads to hospitalization) and reduce costs (avoid seeking help and/or support from others). Although contributing to clinical severity, such traits may not equally contribute to the cost-of-illness of PDs.

The results of the present study could be directly compared to the previous study in the Network of the categorical model of PDs, which used the same sample and the same covariates of other mental health and substance use disorders in the regression models. It investigated the contribution of the specific DSM-5 PD categories on the level of societal costs, and found that no specific type of PD had a unique contribution to the overall level of societal costs [60]. The level of explained variance in the categorical model (*R*^2^ = 0.030) was somewhat higher than in the four different severity models of the present study (*R*^2^ in the range 0.021–0.022), as described in Tables 7, 8, 9 and 10. In sum, none of the four different measures of PD severity seemed to be better predictors of societal costs than the model with PD categories as predictors. On the subcomponent level, BPD was the only significant predictor of health service costs in the categorical model, whereas two of the four severity indicators were significant predictors of health service costs. None of the severity indicators was significant predictors of productivity loss, whereas BPD, avoidant PD, and unspecified PD (mostly composed of BPD and avoidant PD traits) were significant predictors of productivity loss in the categorical model [60]. Thus, the present categorical PD model has a greater sensitivity than two of the severity indicators in predicting the level of health service costs, and a greater sensitivity than all severity indicators in predicting the level of productivity loss.

The inclusion of the five most frequent other mental health and substance use disorders as covariates increased the level of explained variance 2–3 times in all three models (compared with preliminary analyses without these covariates). Even so, the levels of explained variance was rather low in all analyses, but comparable to other PD studies focusing on societal costs [55, 60]. As in models with PD categories [60], having an eating disorder was the only significant covariate in all analyses, with a modest beta weight. The relatively high level of health service costs due to extensive periods of inpatient treatment is the main explanation of this finding.

### Strengths and limitations

A major strength of this study is the high number of participants, with data collected nationwide in different settings (rural/urban, etc.), hence enhancing the external validity of the results. On the other hand, the patients in this study are referred to specialist treatment programs, therefore the results may not be generalizable to all prevalent cases in the community. Another strength of the study is the inclusion of other mental health and substance use disorders as covariates, as most patients with PDs sustain high levels of comorbidity, thus reducing the possibility of confounding variables.

Limited variability of societal costs within the sample of treatment-seeking patients could have led to an underestimation of significance and level of explained variance in these analyses. It is possible that a broader population study could uncover larger variability explained by PD severity. The use of health services data and workforce participation are collected retrospectively, and may be susceptive to recollection bias. The relatively short measurement period of six months was set to reduce this, but the limited range may have reduced the variation in the sample at the same time. This may again have led to reduced levels of explained variance and stability.

Diagnostic reliability was not investigated directly in this study, and inadequate consistency could lead to biased estimators and reduced statistical significance. However, the interviewers had received systematic training in diagnostic interviews and principles, and all diagnoses were set or evaluated by a specialist in psychiatry or clinical psychology. Furthermore, in a former study within the same Network, inter-rater reliability was acceptable [17].

It is a strength that three of the severity indicators, i.e., the number of PDs, the total number of PD criteria, and the number of BPD criteria, as well as the covariates of other mental health and substance use disorders, were based on standardized, semi-structured diagnostic interviews. However, LPFS was assessed by self-report only, by the use of the LPFS-BF 2.0 questionnaire. As it is only a 12-item screening instrument assessing general impairment in personality functioning [69], it will not be able to capture all aspects of personality dysfunction, including externalizing features and aspects such as reality testing and harm to self and others. The limited number of items also means that the instrument captures only a limited degree of variance in the societal costs. Furthermore, patients with severe PD may lack sufficient self-insight to fill out the questionnaire reliably. Thus, in future research, LPFS should be assessed by structured interviews, for instance by the use of SCID-5-AMPD [24] or STiP-5.1 [26]. In addition, it should be noted that the combination of a self-report questionnaire with a structured clinical interview would probably provide more reliable measures than using either self-report or diagnostic interview [23].

## Conclusion

The impact of severity, as measured by the number of PD diagnoses, the number of PD criteria, the number of BPD criteria, and the total score of LPFS-BF 2.0, on societal costs were small and non-significant, and they were not better predictors of societal costs than PD categories, as demonstrated in other studies with categorical PD models. As this is the first study of the impact of PD severity on societal costs, further replication is necessary, possibly including other measures of severity.

## Data Availability

Due to restrictions imposed by the Regional Medical Ethics Committee regarding patient confidentiality, data are available upon request. Requests for data may be sent to the hospital's Privacy and Data Protection Officer at: personvern@ous-hf.no.
